# Endoscopic ultrasound-guided ablation of a metastatic hepatogastric lymph node from esophageal cancer using absolute ethanol

**DOI:** 10.1055/a-2684-4100

**Published:** 2025-09-18

**Authors:** Liliangzi Guo, Cheng Wei, Xiang Cheng, Yang Song, De-Feng Li, Jun Yao

**Affiliations:** 1 12387 Department of Gastroenterology, Shenzhen People’s Hospital (The First Affiliated Hospital, Southern University of Science and Technology; The Second Clinical Medical College, Jinan University), Shenzhen, Guangdong, China

A 78-year-old patient, who had a history of endoscopic submucosal dissection (ESD) for early stage esophageal cancer, had been confirmed as having invasive esophageal squamous cell carcinoma 2 years previously ( Fig. 1 ). They had declined surgical intervention and instead undergone multiple courses of radiotherapy. Unfortunately, a raised lesion was again detected in the esophagus and treated by endoscopic mucosal resection (EMR) ( Fig. 2 ). Histopathologic findings revealed moderately differentiated squamous cell carcinoma with negative margins; however, an abdominal computed tomography (CT) scan identified an enlarged lymph node and positron emission tomography (PET)-CT suggested metastatic involvement in the lymph nodes of the hepatogastric space. Additionally, endoscopic ultrasound (EUS)-guided fine-needle biopsy of the lymph node was performed ( Fig. 3 ), which confirmed poorly differentiated squamous cell carcinoma, consistent with metastatic spread from the esophageal primary lesion ( Fig. 4 **a** ).

**Fig. 1:**
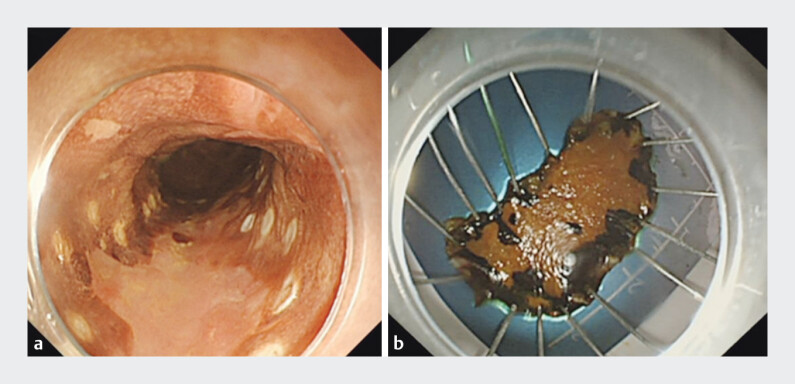
Images of the lesion found and removed by endoscopic submucosal dissection in a 78-year-old patient, which was found to be an early stage esophageal squamous cell cancer.

**Fig. 2:**
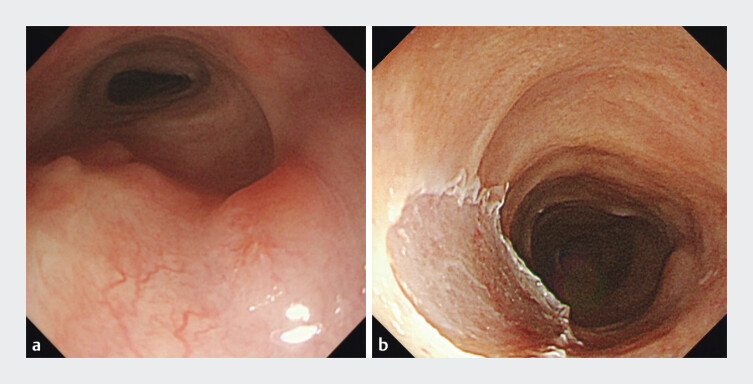
Endoscopic views after the patient had undergone multiple courses of radiotherapy showing: **a** a raised lesion in the esophagus; **b** the appearance after treatment of the lesion with endoscopic mucosal resection.

**Fig. 3:**
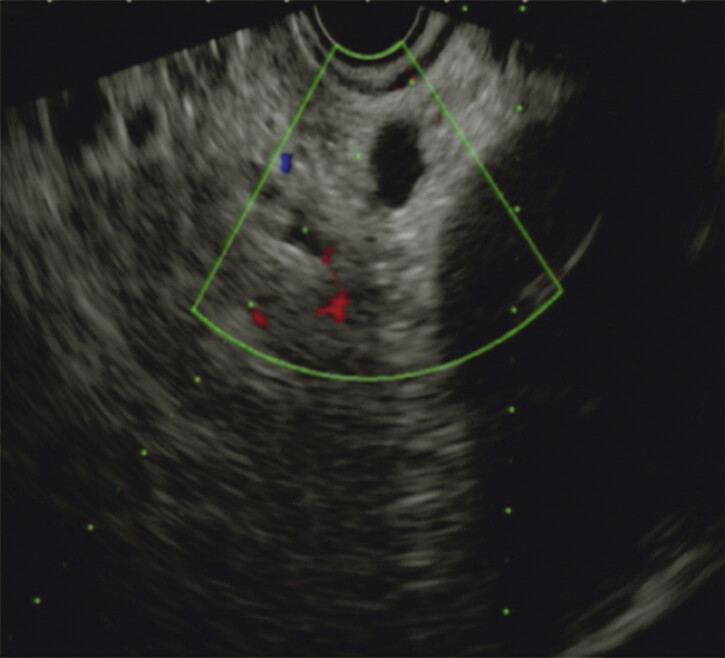
Endoscopic ultrasound image showing an enlarged lymph node in the hepatogastric space.

**Fig. 4:**
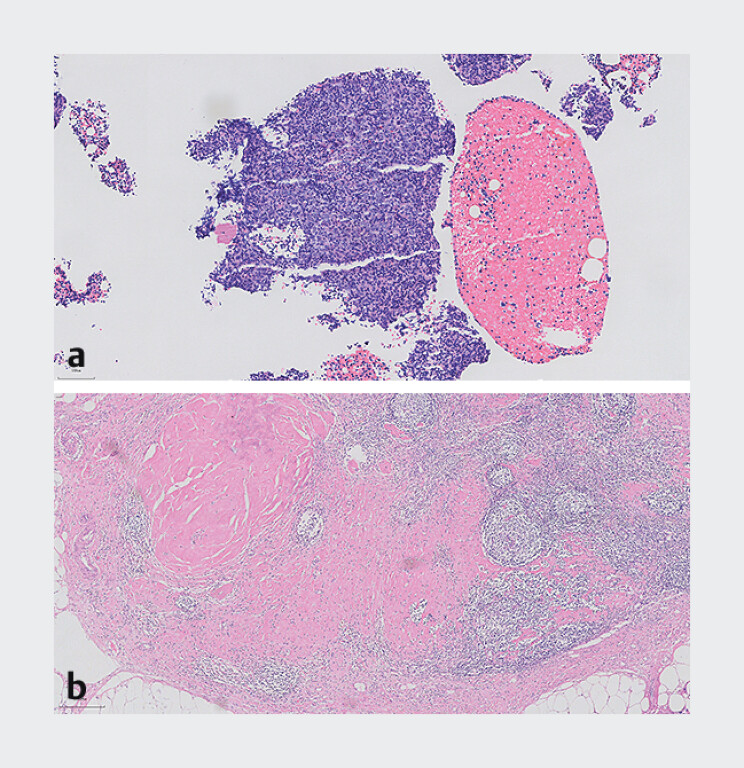
Histopathologic appearance of: **a** the endoscopic ultrasound (EUS)-guided fine-needle biopsy of the lymph node, which was confirmed as being poorly differentiated squamous cell carcinoma; **b** the excised lymph node after the ethanol ablation treatment showing fibrotic changes and collagen deposition.

Given the patientʼs refusal to undergo surgery, a novel treatment was performed. Under EUS guidance, 2 mL of absolute ethanol was injected into the metastatic lymph node to ablate it ( Video 1 ). After 3 months, a repeat abdominal CT scan showed a significant reduction in size of the lymph node. Despite persistently refusing to undergo radical esophagectomy, 5 months later, the patient consented to undergo laparoscopic lymph node dissection. Pathologic examination showed no evidence of malignancy. Notably, the lymph node previously treated with ethanol ablation exhibited fibrotic changes and collagen deposition, indicating successful ablation of the malignant cells ( Fig. 4 **b** ).

A novel application of endoscopic ultrasound-guided ethanol ablation for a metastatic hepatogastric lymph node from esophageal squamous cell cancer.Video 1

This is the first report of EUS-guided ethanol ablation for a metastatic lymph node from esophageal cancer. The procedure resulted in complete collagenization of the lymph node, mimicking the therapeutic effect of surgical lymphadenectomy. The mechanism of ethanol ablation involves direct cytotoxic effects, vascular disruption, and subsequent fibrotic remodeling, which collectively contribute to the effective eradication of tumor cells 1 2 . This innovative approach provides a minimally invasive and alternative option for managing metastatic lymph nodes, and may potentially expand the armamentarium against metastatic disease. Further studies are warranted to validate its efficacy and safety.

Endoscopy_UCTN_Code_TTT_1AS_2AB

## Notes

Endoscopy E-Videos https://eref.thieme.de/e-videos
*E-Videos* is an open access online section of the journal *Endoscopy* , reporting on interesting cases and new techniques in gastroenterological endoscopy. All papers include a high-quality video and are published with a Creative Commons CC-BY license. Endoscopy E-Videos qualify for HINARI discounts and waivers and eligibility is automatically checked during the submission process. We grant 100% waivers to articles whose corresponding authors are based in Group A countries and 50% waivers to those who are based in Group B countries as classified by Research4Life (see: https://www.research4life.org/access/eligibility/ ). This section has its own submission website at https://mc.manuscriptcentral.com/e-videos .

## References

[1] Yu SCH Hui JW Li L Comparison of chemoembolization, radioembolization, and transarterial ethanol ablation for huge hepatocellular carcinoma (≥10 cm) in tumour response and long-term survival outcome Cardiovasc Intervent Radiol 2022 45 172 181 10.1007/s00270-021-02777-6 34604920

[2] Paz-Fumagalli R Li X Smallridge RC Ethanol ablation of neck metastases from differentiated thyroid carcinoma Semin Intervent Radiol 2019 36 381 385 10.1055/s-0039-1696651 31798211 PMC6887524

